# Role of Nano-miRNAs in Diagnostics and Therapeutics

**DOI:** 10.3390/ijms23126836

**Published:** 2022-06-20

**Authors:** Donatella Coradduzza, Emanuela Bellu, Antonella Congiargiu, Aleksei Pashchenko, Evzen Amler, Alois Necas, Ciriaco Carru, Serenella Medici, Margherita Maioli

**Affiliations:** 1 Department of Biomedical Sciences, University of Sassari, 07100 Sassari, Italy; donatella.coradduzza@libero.it (D.C.); ema.bellu@hotmail.it (E.B.); antcong895@gmail.com (A.C.); alexpko@seznam.cz (A.P.); carru@uniss.it (C.C.); 2 Institute of Biophysics, 2nd Faculty of Medicine, Charles University, V Uvalu 84, 150 06 Prague, Czech Republic; amler@seznam.cz; 3 University Centre for Energy Efficient Buildings, Czech Technical University in Prague, Trinecka 1024, 273 43 Bustehrad, Czech Republic; 4 Faculty of Veterinary Medicine, University of Veterinary and Sciences Brno, Palackeho tr. 1946/1, 612 42 Brno, Czech Republic; necasa@vfu.cz; 5 Department of Chemistry and Pharmacy, University of Sassari, 07100 Sassari, Italy; sere@uniss.it; 6 Center for Developmental Biology and Reprogramming (CEDEBIOR), Department of Biomedical Sciences, University of Sassari, Viale San Pietro 43/B, 07100 Sassari, Italy

**Keywords:** nano–microRNA, nanotechnology biomarker, target therapy

## Abstract

MicroRNAs (miRNA) are key regulators of gene expression, controlling different biological processes such as cellular development, differentiation, proliferation, metabolism, and apoptosis. The relationships between miRNA expression and the onset and progression of different diseases, such as tumours, cardiovascular and rheumatic diseases, and neurological disorders, are well known. A nanotechnology-based approach could match miRNA delivery and detection to move beyond the proof-of-concept stage. Different kinds of nanotechnologies can have a major impact on the diagnosis and treatment of miRNA-related diseases such as cancer. Developing novel methodologies aimed at clinical practice represents a big challenge for the early diagnosis of specific diseases. Within this context, nanotechnology represents a wide emerging area at the forefront of research over the last two decades, whose potential has yet to be fully attained. Nanomedicine, derived from nanotechnology, can exploit the unique properties of nanometer-sized particles for diagnostic and therapeutic purposes. Through nanomedicine, specific treatment to counteract only cancer-cell proliferation will be improved, while leaving healthy cells intact. In this review, we dissect the properties of different nanocarriers and their roles in the early detection and treatment of cancer.

## 1. Introduction

Early detection and effective treatments are crucial for disease eradication and patient healing, and for survival and/or quality of life. Cancer is one of the pathologies that can benefit from both approaches, as many other illnesses. MicroRNAs (miRNA) represent an emerging tool which could be effective in either detecting or curing several conditions. miRNAs are short (18–25 nucleotides) noncoding single-stranded endogenous oligoribonucleotides, which are relatively stable; they are predominantly secreted in vesicles, or in a complex with other proteins. MiRNA’s role is linked to the regulation of different biological processes, such as cellular development, differentiation, proliferation, metabolism, and apoptosis [1]. In fact, they regulate gene expression, and the alterations in their expression levels correlate with the onset and progression of different diseases, such as tumours, cardiovascular and rheumatic diseases, and neurological disorders [2,3,4]. Under pathological conditions, dysregulated miRNA levels are observed, but the relationship between dysregulated miRNA levels and disease is not straightforward. The oncogenic or tumour-suppressive activities of miRNAs depend on which genes are activated or inhibited through the up- or downregulation of miRNA expression. Thus, to ensure a secure diagnosis, the identification of miRNAs via expression profiling is fundamental; this is because alteration in the expression levels of a single miRNA does not have sufficient diagnostic power compared to multiplexing, that is, the parallel or simultaneous detection of known multiple miRNAs. Current quantification strategies have limitations and disadvantages. In general, all quantification methods are divided into two categories—one that utilizes direct oligo-hybridization without sample RNA amplification, and the other requiring sample amplification. Methods that do not utilize sample amplification will require a relatively larger starting amount of total RNA, while the others requiring sample amplification, with external variation as they are handling imperfections, can also be amplified. Oligo-microarray technology is relatively low-cost and readily available; a disadvantage of this method is its scale, as the resulting array will be relatively large. Alternatively, the use of synthesis and chemical modification of RNA probes is costly and often requires a large amount of total RNA. Many other methods and tools have been developed for miRNA expression profiling. Problems such as sensitivity and specificity have been addressed through various strategies; however, they are still very expensive, since answering to a variety of specific needs; an appropriate sample size, sample quantity and speed; and the requirement to identify new miRNAs can be costly. Thus, there is a great interest in developing innovative methods, and nanotechnology-based approaches are particularly sought-after. Nanotechnology will have a strong impact on delivery and diagnosis through miRNA, demonstrating that the newly developed approach works on ‘real-world’ samples under standardized conditions. The same is true also for miRNA transport and delivery, as miRNA inhibition or mimicry are strategies currently under evaluation to maintain the level of miRNA inside the cells, and nanotechnology can offer a good solution to bring the miRNA mimics to the tumour site.

**Figure 1:**
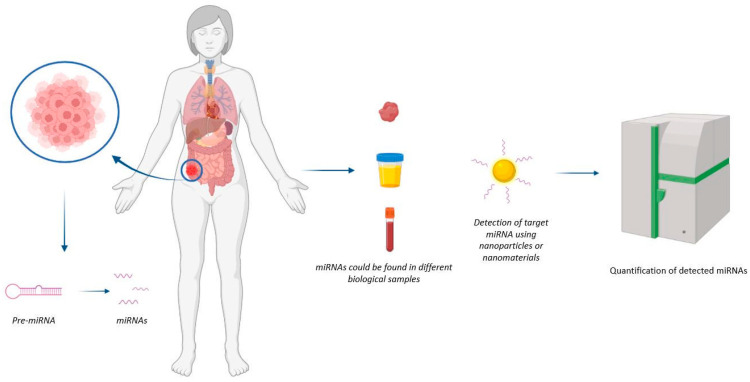
Representation of mechanism of action of nanodevices for miRNA detection. The nanodevice can detect the miRNA of interest at the tumour site.

**Figure 2:**
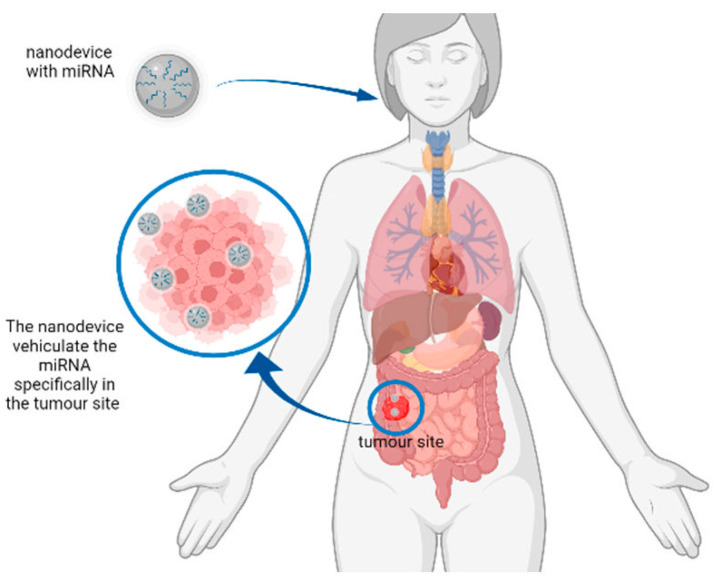
Representation of a hypothetical mechanism of action of nanodevices for miRNA delivery. The nanodevice can deliver the miRNA of interest to the tumour site to reduce the expression of tumour-related genes.

In this review, both approaches (detection and treatment) using nanotechnology-based strategies have been analysed, to point out the current trends in this promising field of bio-medical applications (Figure 1 and Figure 2).

### MiRNA Detection and Biosensing

Conventionally, miRNA detection is based on real-time polymerase chain reaction (qRT-PCR), microarrays, Northern blotting, and Next-generation sequencing (NGS). These measurement methods just reflect the average gene expression level and cannot provide the heterogeneity and transient spatiotemporal variations of miRNAs in living cells [5]. The main difference between nanotechnology-based and conventional methods lies in the transduction mechanism. The peculiar physicochemical properties of nanostructured materials are essential to enhancing signal readout [6]. Several nanoparticles (NPs) have excellent optical properties, making them ideally suited for the development of sensing strategies. Some NPs are bright and stable fluorescence emitters, such as silver nanoclusters (AgNCs) and quantum dots (QDs), and can be used either directly or in fluorescence resonance energy transfer strategies. Other NPs, such as gold NPs (AuNPs) and carbonaceous NPs, can be used as efficient fluorescence quenchers in fluorescence recovery approaches. In the presence of target miRNA, the fluorophores, physically or chemically released from the NP, can emit fluorescence in a concentration-dependent manner.

These few examples indicate that nanomaterials can have great potential in miRNA detection and biosensing. Biosensors represent innovative analytical tools for clinical diagnosis as well as for a better understanding of the molecular mechanisms involved in pathophysiology, revealing new biomarkers that are useful for the evaluation of appropriate pharmaceutical treatments [7]; moreover, miRNAs are surely promising and effective biomarkers. Biosensors can help in the early diagnosis and monitoring of pathological conditions, particularly for oncological diseases, and are useful in prognosis, surveilling the evolution of the disease, and opening the door to access to global health care. Among the different biosensing techniques, plasmonic sensor platforms are able to analyse different classes of biomolecules of clinical interest [8]. Different classes of biomolecules can be quantitatively detected in real time via high-throughput exploitation of (Localized) the surface plasmon resonance (SPR or LSPR) of metal nanoparticles and nanofilms, able to monitor and also perform label-free interactions. In fact, surface plasmons have been employed to enhance the surface sensitivity of different spectroscopic techniques, such as fluorescence and Raman scattering, applied to the detection of biomolecules. In the case of metal nanoparticles, LSP oscillations are responsible for the deep colors of their suspensions or sols, due to strong absorption bands in the ultraviolet–visible region, that are not present in the bulk material. The surface interaction of metal nanoparticles with biopolymers such as proteins, DNA, and RNA causes shifts in this resonance that can be used to detect and quantify their presence.

Herein, we review the progress made over the past five years in SPR and LSPR-based platforms for the detection of several cancer biomarkers in liquid biopsy samples. Thus, we will mainly focus on recent applications of SPR-based platforms to detect and quantify biomarkers in biofluids collected directly from cancer patients. Finally, we describe the analytical performance of selected SPR biological assays and their significant advantages in terms of high sensitivity and specificity, as well as accuracy and simplicity of workflow, for their future application in clinical practice. The discussed contributions were selected by evaluating analytical performance in terms of sensitivity and specificity, and accuracy, not only in the swab but, above all, in biological fluids, for future applications in clinical diagnosis [9,10].

## 2. Outcome

### 2.1. SPR-Based Colorimetric Biosensors

Colorimetric biosensors are used to detect a particular analyte through color changes easily detected by the naked eye or using simple portable optical detectors for quantitative measurement. The direct, label-free transduction of biomolecular interactions into a physicochemical signal that can be easily and sensitively quantified in real time is critical to applications in clinical diagnostics, the real-time detection of environmental and biological toxins for field-use, and in proteomics [11]. Various approaches to exploit advances in nanoscience and nanotechnology for bioanalysis have recently been reported, as the thrust in biosensor research moves towards miniaturization and multiplexing, so as to simultaneously and rapidly detect a large number of analytes in ever-smaller volumes, to increase sensitivity and throughput and reduce cost.

A summary of existing and emerging techniques that can be employed to detect miRNA through a colorimetric assay design with a detailed discussion of their sensing principles, performances, as well as pros and cons, as proposed here; this can provide guidelines on the selection of suitable colorimetric biosensors for detecting different miRNA in cancer.

Hamidreza Mollasalehi et al. (2021) developed a colorimetric nanobiosensor based on non-crosslinking Au-nanoprobes without an amplification requirement for the detection of miR-21 and miR-155, two circulating microRNA biomarkers that are upregulated in prevalent cancers. The upregulated miRNAs in cancerous cell lines provided significant colorimetric variation in the Au-nanoprobe absorbance spectrum while the samples isolated from normal cells did not show any colorimetric variation. The limit of detection (LOD) of the method was determined to be less than 1 ng/µL of total isolated miRNA. This method is an easy-to-perform technique without the need for sophisticated equipment that could be applied to cancer detection with high sensitivity and specificity [12].

MiR-141 is a promising biomarker for human prostate cancer [13,14,15]. This miRNA has been identified with high selectivity in human serum with a LOD of ca. 0.48 nM. Park Yeonkyung et al. (2018) developed a label-free, enzyme-free, colorimetric method for the detection of different miRNAs based on a target-catalysed toehold-mediated strand displacement (TMSD) reaction. In the presence of target miRNA that binds to the target-recognition domain of a detection probe, a catalyst strand is released to trigger the TMSD reaction, resulting in the generation of an active G-quadruplex DNAzyme.

It was possible to use this method for the analysis of different miRNAs by simply redesigning the detection probe while retaining the TMSD-based signal transduction module. In this article the applicability of the method was evaluated by detecting another target miRNA, let-7d, known to be downregulated in many cancers including head and neck, pancreatic, ovarian, and lung cancer. let-7d was identified with a LOD of ca. 1.06 nM [16,17].

To detect miRNA-148a, a promising biomarker for the early diagnosis of gastric cancer, Ye et al. (2017) used an SPR colorimetric biosensor that was able to sense this miRNA directly inside the cancer cells. In an assay system, the sensing probes are facilitated by the conjugation of AuNPs with RNA probes (RNAP). When miRNA-148a is present, the AuNPs-RNAP conjugates and targets are able to initiate the sandwich hybridization reaction, resulting in changes in the SPR absorption band, microscopic distribution, and macroscopic color of the AuNP solution. The LOD of the test was ~1.9 nM, with high sensitivity and selectivity of the biosensor [18].

Cai et al. (2020) developed a simple and low-cost colorimetric method for the quantitative detection of miRNA-148a, a promising biomarker for the early diagnosis of gastric cancer. Mir-148 was detected due to the unique SPR absorption of the AuNPs employed. The detection of the sensing probes is facilitated by the conjugation of AuNPs with RNA probes (RNAP) via Au–S bonds, which align in a tail-to-tail fashion onto the target RNA. A sandwich hybridization reaction is triggered between the AuNP–RNAP conjugates and miRNA-148, resulting in changes in the SPR absorption band, microscopic distribution, and macroscopic color of the AuNP solution. The proposed colorimetric biosensor can detect miRNA-148a down to the limit of a ~1.9 nM concentration [19].

### 2.2. Other SPR-Based Biosensors

Among the different biosensing techniques, plasmonic sensor platforms are able to analyse different miRNAs of clinical interest [8]. The performance of the SPR technique to monitor label-free interactions and to quantitatively detect biomolecules in real time with high throughput have marked its role in clinical diagnosis. Generally, SPR sensing can examine the interactions between biomolecules based on affinity-binding analysis, including antibody–antigen interactions [20], ligand-receptor kinetics [21,22], enzyme–substrate reaction (Massumi Miyazaki et al., 2017), and epitope mapping [23]. In this section, we examine the advances made over the last five years in SPR for the detection of different miRNA cancer biomarkers.

Several miRNA biosensors are based on the association of graphene oxide and gold nanoparticles. Wang et al. (2016) made their contribution by developing a sensitive SPR biosensor for miRNA detection using graphene oxide–gold nanoparticle (GO–AuNPs) systems for signal amplification. An Au film surface was employed to immobilize the thiolated NP DNA-capture probe with a short, complete complementary sequence to recognize the part sequence of target miRNA. Following this, the assistant DNA-linked GO–AuNP hybrids were used to bind the other section of the target. In this way the SPR biosensor was able to reach a detection limit as low as 1 fM. The GO–Au-SPR was used for the detection of miRNA-141 in cell extractions and showed superior sensitivity and excellent selectivity in discriminating between the five miRNA-200 family members [24]. Additionally, Li et al. (2017) developed a biosensor based on two layers of graphene oxide–gold nanoparticles (GO–AuNPs) for the detection of miRNA-141 and adenosine. The immobilization of the DNA-capture molecule was carried out in the bottom layer, acting as a functionalized substrate on the sensor chip, while the upper layer worked as a signal-amplification element. Such an amplification strategy determined the impressive sensitivity of this new SPR biosensor, which reached a detection limit of 0.1 fM in the measurement of miRNA-141, together with an appreciable selectivity toward miRNA-200 family members. In addition, it could be applied to complex systems, such as in cancer cell and serum, and especially demonstrates its applicability for the detection of miRNA-141 in cancer-cell extractions. The results obtained were consistent with those obtained using qRT-PCR. Finally, the system was able to detect adenosine using this SPR biosensor combined with a split aptamer [25].

Li et al. (2016) developed a surface SPR biosensor for the detection of target miRNA, exploiting the mismatched catalytic hairpin assembly (CHA) amplification coupled with programmable streptavidin aptamers (SA-aptamers). The allosteric effect of CHA amplification was produced by the presence of target miRNA, which triggered the recycling of the target miRNA and yielded large quantities of CHA products, activating SA-aptamers. The abundant CHA products were able to hybridize with the capture probes on the sensor-chip-activated SA-aptamers and caused an increase in and output of the detection signal. This system was able to detect target miRNA down to 1 pM with a dynamic range from 5 pM to 100 nM. This method was tested on the determination of target miRNA spiked into human total RNA samples with great success [26].

Liu et al. (2017) proposed an SPR biosensor for the detection of miRNAs and cancer cells based on a multiple-signal amplification procedure. Additionally, in this case, a gold film was used to immobilize a thiol-modified hairpin probe, with a sequence complementary to the target miRNA. When it encounters the target miRNA, the stem-loop structure of the hairpin probe is unfolded, so the terminus of the latter is hybridized with DNA-linked AuNPs. These, in turn, start the formation of a DNA “supersandwich” structure due to the addition of two report DNA sequences. A further enhancement in the SPR response was obtained through the absorption of positively charged silver nanoparticles (AgNPs) onto the long-range DNA surpersandwich. This amplification strategy resulted in a detection limit of 0.6 fM for miRNA-21 and high selectivity toward single-base mismatches [27].

Another biosensor based on gold-nanoparticle-decorated molybdenum sulfide (AuNPs-MoS_2_) was proposed for miRNA detection by Nie et al. (2017). The strategic design includes the usual gold film to which thiol-modified DNA oligonucleotide probes, a sequence complementary to the target miRNA-141, were fixed in order to identify the segment sequence of the target miRNA. Then, the other section of the miRNA-141 was bonded by assistant DNA-linked AuNPs-MoS_2_ nanocomposites. In this way the new biosensor was able to detect miRNA with high selectivity and a LOD of 0.5 fM. The method was also highly specific, and was able to distinguish differences among miRNA-200 family members [28].

An SPR sensor integrating DNA tetrahedron probes (DTPs) onto gold films (DTPs-Au) was developed by Nie et al. (2018) and applied in the detection of miRNA in a complex matrix with high sensitivity. This SPR sensor exploits the amplification of the catalytic growth of AuNPs, shows excellent selectivity, and can distinguishbetween homologous miRNAs of the let-7 family. The sensor showed a detection limit of 0.8 fM for let-7a, and a sensitivity that could be used for detecting miRNA in undiluted human normal serum and cell lysate [29].

Recently, Portela et al. (2020) fabricated a nanoplasmonic sensor chip based on nanogap antennas by employing a colloidal lithography process. The nanostructured plasmonic sensor chip has been used for the detection of miRNA-210, an important biomarker for lung cancer diagnosis, via a DNA/miRNA hybridization assay. The LOD was of 0.78 nM [30]. Li et al. (2019) developed self-assembling nanostructures with controllable plasmonic functionalities for miRNA detection in bioanalysis applications through the use of broad surface-enhanced Raman scattering (SERS). In this case, miRNAs both work as cancer biomarkers and triggers of direct self-assembly, leading to their detection. In fact, due to the precise nucleic acid hybridization of miRNA targets, Au nanospheres are able to selectively self-assemble onto hollowed Au/Ag alloy nanocuboids. Such nanostructures possess the proper interparticle distances (≈2.3 nm) for optimal SERS signalling, and further increase miRNA detection results through nanozyme catalytic SERS signalling. The resultant self-assembled nanostructures provided miRNA detection in the fM range, together with high sensitivity and specificity, for the accurate detection of miR-107 [31].

### 2.3. Graphene-Based Biosensors

In recent years, graphene has attracted enormous attention in different research fields due to its excellent electrochemical properties, adsorption performance, mechanical strength, and flexibility. For these many reasons, it can also be an attractive candidate to prepare effective biosensors [32], as previously seen in a couple of examples wherein it was used in combination with gold films or nanoparticles (*vide supra*). The remarkable properties of graphene and graphene derivatives make them ideal in the formulation of new biosensors with high sensitivity and low detection limits, thanks to their outstanding sensing abilities (e.g., high specific surface area, remarkable electronic properties, superlative flexibility, and electron-transport capabilities). Thus, impressive work has been carried out in the most recent years to prepare graphene-based biosensors for quantitative detection of cancer-related biomarkers such as miRNA.

Treerattrakoon et al. (2019) exploited rolling circle amplification and the fluorescence-quenching properties of reduced graphene oxide to develop a multiplex miRNA-detection platform. Two fluorescence-labelled circular ssDNA tags were added to the system for the detection of miR-29a and miR-144, which are particularly important since they are highly expressed in some patients with cancer. The resulting platform showed high sensitivity and selectivity, with a LOD of 0.05 pmol, and was able to detect multiplex miRNAs from both human serum and HeLa cells [33].

An electrical sensor that enables direct identification of miRNAs in patient urine was developed by Kim et al. (2021). The proposed sensing platform used a disposable electrical sensor chip consisting of a reduced graphene oxide nanosheet in which the sensing module is simply connected to an FET body. Such a platform provided a method for the real-time detection of miRNAs in patient urine, without pre-treatment or signal-amplification steps. The sensor showed high specificity and sensitivity, allowing the detection of target miRNAs in a broad dynamic range within 20 min in human urine specimens, while enabling simultaneous quantification of multiple miRNAs with a detection limit as low as 10 fM. This is particularly useful in a complementary strategy for improving the accuracy of testing for the early diagnosis of prostate cancer, thus combining standard serum PSA with urinary miRNA tests before a biopsy is taken [34].

### 2.4. Carbon-Quantum-Dot-Based Biosensors

Carbon quantum dots (CQDs) [35] are novel zero-dimensional carbon-based nanomaterials with strong fluorescence characteristics. They are monodispersed spherical nanoparticles with a carbon-based skeleton and a large number of oxygen-containing groups on the surface [36]. CQDs not only possess the excellent optical properties of traditional semiconductor quantum dots, but also low cytotoxicity and low environmental and biohazard risk with respect to the traditional materials. Moreover, they show excellent water solubility and photostability. Therefore, they represent very good candidates in the search for effective and sensitive sensors for miRNA detection and quantification.

A FRET-based platform (FRET = Förster or fluorescent resonance energy transfer; thus, a fluorometric method) for the sensitive detection of miRNAs was developed by Mohammadi et al. (2018). Carbon dots (C-dots) were loaded with a DNA probe as the fluorophore, and MnO_2_ nanosheets as the quenching agent. The MnO_2_ nanosheets are able to quench the blue–green fluorescence of the DNA-loaded C-dots, but the binding of target miRNA-155 would restore it. The correlation of fluorescence with the log of miRNA-155 concentration was linear in two ranges, i.e., 0.15–1.65 aM and 1.65–20 aM, and LOD was found to be 0.1 aM. The assay discriminates between fully complementary and single-base-mismatch miRNA. It displayed high specificity when used to detect MCF-7 breast cancer cells in concentrations from 1000 to 45,000 cells/mL, with a 600 cells/mL LOD. The method obtained satisfactory results when applied to the analysis of serum samples spiked with microRNAs [37].

On a similar basis, a signal-on FRET nanobiosensor obtained with a carbon-quantum-dot (CQD)-labelled molecular beacon, was applied to the detection of intracellular miRNA-21 by Mahani et al. (2019). The molecular beacon (MB) is a hairpin-shaped oligonucleotide hybridization probe with an internally quenched fluorophore whose fluorescence is restored when it binds to a target nucleic acid sequence such as miRNAs, and in this case, it was labelled with the CQDs at the 5′ end, and with Black Hole Quencher 1 (BHQ1) at the 3′ end. The two labels, thus, behave as the donor and acceptor parts of a FRET system. In the presence of miRNA-21, the fluorescence intensity of the CQDs at 460 nm (excitation at 360 nm) is restored; upon hybridization with miRNA-21, the hairpin structure of the MB opens up its loop, increasing the distance between the BHQ1 quencher and the CQDs and leading to fluorescence changes. The probe is highly specific and sensitive, with a LOD of 0.3 nM. It is able to distinguish between miRNA-21 and its single mismatch mutant; thus, it represents a precious tool for early cancer diagnosis [38].

Mohammadi et al. (2021) prepared carbon-dot–chitosan nanocomposite hydrogels for the detection of miRNA-21 in breast cancer cells (MCF-7). The synthesis of three fluorescent hydrogels was carried out via the Schiff base reaction of the amine group on chitosan, with the aldehyde groups present on the carbon dots’ surface, and functionalization with an ssDNA probe. The DNA hydrogel biosensors showed high stability and a superb sensitivity for miRNA-21, with a suitable linear range between 0.1–125 fM, and a LOD of 0.03 fM [39]. Another strategy for the detection of miRNA was used by Kuntip et al. This was based on miRNA adsorption on nanomaterials and dissociation by a complementary DNA strand (DNA probe). In this work, graphene quantum dots (GQDs) were used as nanomaterials for miRNA adsorption. MiR-29a, a potential cancer biomarker, was used as a model for a thorough study on the mechanism of the adsorption process and the interactions between this miRNA and the GQDs [40].

### 2.5. Chitosan-Molecular Beacon (CS-MB)

As shortly mentioned above, molecular beacon (MB) technology is used to detect DNA/RNA expression in living cells. Nanoparticles of chitosan (CS), a natural polymer, can be employed in this field due to their low immunogenicity and toxicity, with high biocompatibility. The positively charged CS nanoparticles bind to the negatively charged molecules such as DNA or small RNA, and condense them into core-shell nanoparticles. This explains miRNA adsorption on nanomaterials and its successive dissociation by a complementary DNA strand (DNA probe).

Multiple exosomal miRNAs were detected via a sensitive detection method based on rolling-circle amplification (RCA) and with the aid of molecular beacons. This technique was used by Wang et al. (2021) to simultaneously replicate multiple exosomal miRNAs from different cell lines, which were then detected by specifically designed molecular beacons (MBs). Normalized fluorescence intensities of MB were applied to measure the relative concentrations of target miRNAs (miRNA-21, miRNA-122, and miRNA-155), which were, in turn, employed to distinguish the breast cancer exosome from a normal one, helping in early cancer diagnosis [41].

Kim et al. (2021), presented a novel molecular-beacon-equipped DNA nanostructure immobilized on a single bead for the detection of a cancer miRNA that was temperature-insensitive. This system, as just mentioned, was composed of three moieties: a molecular beacon for cancer-specific RNA capture, a stem body as the core template, and a single bead for solid support. This system is able to bind a given target miRNA marker and simultaneously provide quantitative indication of probe internalization that can function as a part of a target-label-free optical genetic biosensor to detect miRNAs [42].

### 2.6. Electrochemical Biosensors

Electrochemical biosensors are sensing devices based on the transduction of biochemical events into electrical signals.

The electrode is a fundamental component, also working as a solid support for the immobilization of biomolecules and electron movement. The current electrochemical biosensors are divided into two groups: carbon-based (carbon nanotubes and graphene) and non-carbon-based nanomaterials (metallic and silica nanoparticles, nanowires, indium tin oxide, organic materials). These biosensors are promising for meeting sensitivity requirements along with demonstrating high reproducibility, particular in reducing the matrix interference from real samples.

Among the firsts, Azimzadeh et al. (2016) developed an electrochemical nanobiosensor for miR-155 detection in plasma based on a hybrid system. This was prepared by layering a graphene oxide (GO) sheet on the surface of the glassy carbon electrode (GCE) and decorating it with thiolated probe-functionalized gold nanorods (GNRs). The electrochemical signal generated by this system was linearly related to the concentration of the target miRNA in the range 2.0 fM-8.0 pM, with a detection limit of 0.6 fM. The nanobiosensor showed high specificity and was able to sharply discriminate between complementary target miRNA; single- and three-base mismatch; and non-complementary miRNA [43].

In a similar way, the detection of miR199a-5p, an early biomarker for triple-negative breast cancer in serum, was developed by Ebrahimi et al. (2018), through an electrochemical nanobiosensor. Additionally, in this case, the fabrication scheme of the biosensor was based on a modified glassy carbon electrode (GCE) covered with graphene oxide (GO) and thiolated probe-functionalized gold nanorods (GNR). In optimal conditions, the linear range of the calibration curve was 15 fM-148 pM, with a LOD of 4.5 fM, and a standard deviation of 2.9%. This nanobiosensor had a high specificity and sensitivity for miR-199a-5p [44].

A different concept was applied to the design of an electrochemical DNA biosensor to be used in the detection of the miRNA of the breast cancer 1 gene mutation (BRCA1). The sensor was based on a double signal amplification (DSA) strategy. In the presence of the target miRNA of BRCA1, the RNA–DNA duplexes formed were precisely recognized by double-strand specific nuclease (DSN), which selectively cleaved the DNA portion of the duplexes while miRNAs were released to initiate another subsequent cycle. In this way the cyclic enzymatic signal boost (CESA) produced a primarily amplified signal. Then, the cleaved DNA probe was selectively hybridized with a triple-CdTe quantum-dot-labelled DNA nanocomposite (3-QD@DNA NC) on the electrode and gave rise to multiple amplified signals. The biosensor, developed by Cheng et al. (2019), showed high sensitivity for the detection of the miRNA of BRCA1 in concentrations ranging from 5 aM to 5 fM, and had a detection limit of 1.2 aM [45].

Zhou et al. (2020) designed an ultrasensitive electrochemical biosensor based on a dual-amplified strategy for the detection of ultra-trace microRNA-141, which exploited the action of a nuclease, as in the previous case. This tool is formed of two split sequences that are G-quadruplex-connected and complementary with miRNA-141 via click-chemistry-mediated nucleic-acid-strand self-assembly. The formed DNA–RNA hybrid duplexes are specifically recognized by the double-strand-specific nuclease (DSN) that cleaves the DNA portion of the duplexes to release the miRNA-141, which initiates the next cycle to acquire primal signal amplification via enzyme-assisted target recycling (EATR). Au electrodes are modified with functionalized fullerene nanoparticles (FC60) with a larger surface-active site and better biocompatibility. These were designed to produce a multiply enhanced amplified signal. This system showed remarkable analytical performance for the detection of miRNA-141 in concentrations ranging from 0.1 pM to 100 nM and with a detection limit of 7.78 fM [46].

A different approach was chosen by Hakimian et al. (2020) who developed an electrochemical biosensor in which a thiolated probe was immobilized on the gold electrode surface for the detection of miR-155, a breast cancer risk factor. When the target miR-155 reached the probe, it formed a negatively charged nanohybrid which was able to absorb positively charged polyethylenemine–silver nanoparticles, as electroactive labels. Therefore, the oxidation of silver nanoparticles produced an anodic peak current which was recorded as the electrochemical signal. The new biosensor provided an ultrasensitive method for the detection of miR-155, with a detection limit of 20 zmol [47].

The same miR-155 was detected by Yazdanparast et al. (2020) using an electrochemical nanobiosensor based on magnetic Fe_3_O_4_NPs@Ag core-shell nanoparticles applied onto the magnetic bar carbon-paste electrode, to which an amine-modified anti-miR-155 was applied, and upon hybridization with target miR-155. Resveratrol (RSV) was eventually applied as an electrochemical label on the double-strand oligonucleotide. Differential pulse voltammetry (DPV) of the oxidation peak of RSV was assumed as the final signal. The linear range of the novel nanobiosensor was 0.5 fM–1.0 nM, and the detection limit was 0.15 fM. The nanobiosensor showed reproducibility and specificity using different types of mismatched target sequences. To confirm that the nanobiosensor was detecting miR-155 without any significant interference from other molecules, real spiked samples of human serum were used [48].

Pothipor et al. (2021) developed a label-free electrochemical biosensor to detect miRNA-21, another biomarker of breast cancer. The sensor was built onto a screen-printed carbon electrode (SPCE) modified with a nanocomposite of graphene (GP), polypyrrole (PPY), and gold nanoparticles (AuNPs), to enhance the electron-transfer properties and increase the degree of methylene blue (MB) intercalation for signal amplification. The GP/PPY-modified electrode showed good electrochemical reactivity and excellent sensor performance. The redox current of MB distinctly increased only in the presence of the miRNA-21 target, due to the formation of the double-stranded miRNA-21/DNA-21 complex. The peak current of the MB redox process, which was proportional to the miRNA-21 concentration on the electrode surface, was monitored via differential pulse voltammetry (DPV). The new biosensor showed a linear range from 1.0 fM–1.0 nM with a LOD of 0.020 fM, with good selectivity, high stability, and satisfactory reproducibility [49].

The same authors proposed a label-free multiplexed electrochemical biosensor to simultaneously detect different miRNAs connected to breast cancer. The sensor was based on a gold nanoparticle/graphene quantum dots/graphene oxide (AuNPs/GQDs/GO) modified three-screen-printed carbon electrode (3SPCE) array. Three redox species are used as redox indicators for anchoring capture miRNA probes, which, in turn, hybridize with the complementary targets, miRNA-21, miRNA-155, and miRNA-210. In the presence of the three targets, the square-wave voltammetry (SWV) scan displays three well-separated peaks. Each peak indicates the presence of one miRNA, and its intensity quantitatively correlates with the concentration of the corresponding target. The biosensor was shown to have high sensitivity and selectivity, multiplexing capability, and it is promising for improving diagnostic accuracy for early breast cancer detection [50].

Following these results, the same authors developed another electrochemical biosensor capable of detecting two different kinds of breast cancer biomarkers simultaneously: cancer antigen 15-3 (CA 15-3) and microRNA-21 (miRNA-21). The sensor is formed by a poly(3-aminobenzylamine)/two-dimensional (2D) molybdenum selenide/graphene oxide nanocomposite-modified two-screen-printed carbon electrode array (dual electrode), each functionalized with 2,3-diaminophenazine-AuNPs and toluidine blue-AuNPs. The redox probe-gold nanoparticles have the dual role of signalling molecules and supporting the immobilization of anti-CA 15-3 antibodies and the capture of DNA-21 probes. Efficient duplex detection is obtained thanks to the good conductivity and high surface-to-volume ratio of the nanocomposite, so that high numbers of the antibodies and capture probes can be immobilized on the modified dual-electrode. The proposed assay strategy reveals excellent analytical performance, and the biosensor can perform the analysis at the femtomolar level (1.2 fM) for miRNA-21 and with a LOD of 0.14 U/mL for CA 15-3. The biosensor provides not only a fast analytical response time, but also simplicity in preparation, high selectivity, reproducibility, and acceptable stability [51].

### 2.7. FRET for miRNA Detection

Förster or fluorescence resonance energy transfer (FRET) has already been introduced in the previous sections (*vide supra*). This effect, occurring between fluorophores of the same species, was recognized in the early- to mid-1900s, and since then, it has been widely used as a spectroscopic technique in all applications of fluorescence, including medical diagnostics, DNA analysis, optical imaging and for various sensing properties. Fluorescence-based sensors adopt three different strategies: (a) fluorescence quenching (turn-off), (b) fluorescence enhancement (turn-on), and (c) fluorescence resonance energy transfer (FRET). FRET sensors are useful for studying intracellular processes. The FRET technique allows the determination of the approach between two molecules within several nanometers of each other; therefore, it has been used to investigate interactions at the molecular level and employed to determine the conformation and structure of proteins; for the detection of the spatial distribution and assembly of proteins; to design biosensors; for nucleic acid hybridization; and for the distribution and transport of lipids [52].

An integrated and self-powered DNA nanodevice loaded on FRET flares, through orderly assembly, is able to automatically amplify signals after being triggered by miRNA-21 in specific cancer cells. The AS1411 aptamer is employed to target specific cancer cells and facilitate cell internalization of the assembly of DNA nanostructures. Li et al. (2021) demonstrated that the technique enabled rapid response to miRNA-21 and improvement of the detection sensitivity compared to previously proposed FRET flares without amplification [53].

A microRNA detection method was developed by Wang et al. (2018) using bright fluorescent quantum dots (QDs), simple DNA probes, and the enzyme duplex-specific nuclease. An isothermal target-recycling mechanism was employed, wherein a single miRNA target prompted the cleavage of many DNA signal probes. The incorporation of DNA-functionalized QDs enabled a quantitative fluorescent readout, mediated by FRET. The system is based on the interaction with the DNA signal probes and achieved highly sensitive detection with a limit of 42 fM (or 1.2 amol) for miR-148, with excellent selectivity versus mismatched sequences and other miRNAs. The method was successfully employed using an alternative FRET pair for the quantification of miR-21 in RNA extracts from human cancer- and normal-cell lines [54].

### 2.8. Nanoflares

Nanoflares are spherical gold nanoparticles densely functionalized with a monolayer of single-stranded DNA (ssDNA) containing a 3′ thiol derivative that is complementary to mRNA for a target gene that allows live-cell detection of intracellular mRNA. The ssDNA “recognition sequence” is prehybridized to a shorter DNA complement containing a fluorescent reporter (the “reporter flare”). Target mRNA binds the recognition sequence, and the reporter flare strand is displaced, to provide a fluorescent readout, which is quenched based on its proximity to the gold particle. Nanoflares, coupled with flow cytometry, are used to fluorescently detect cancer biomarkers in the context of whole blood. Nanoflares provide the first genetic-based approach for detecting, isolating, and characterizing live cancer cells from blood, and may provide new opportunities for diagnosis, prognosis, and personalized therapy of cancer.

Li et al. (2018) presented two-color-based nanoflares for simultaneous detection of two different miRNAs—miR-21 and miR-141—expressed in high levels in various live cancer cells. The nanoflares are AuNPs functionalized with a thick shell of recognition sequences hybridized to two short fluorophore-labelled DNA molecules. When target miRNAs bind the recognition sequence, the associated displacement of the flare can be detected as a corresponding increase in fluorescence. In this conformation, the close proximity of the fluorophore to the AuNP surface leads to the quenching of the fluorescence; this permits the study of the two-color-based nanoflares and simultaneous detection of both miRNAs [55].

To detect exosomal miRNAs in situ, Zhao et al. (2020) proposed a thermophoretic sensor implemented with nanoflares. Thermophoretic accumulation of nanoflare-treated exosomes is able to cause an amplified fluorescence signal upon the binding of exosomal miRNAs to nanoflares. This strategy allows for direct and quantitative measurement of exosomal miRNAs, and is able to profile exosomal miRNAs down to 0.36 fM in 0.5 μL serum samples, without requiring RNA extraction or target amplification [56].

To discriminate between target cancer cells, even in a mixed-cell culture system, Qing et al. (2020), developed a biosensor using AuNPs deposited with a thin layer of platinum; this enabled them to detect miRNA-21 in living cells with the capability of completely discriminating diseased target cells, even in a mixed-cell system. Compared to previous methods, changing the ligand chemistry is more accessible with this system, which is more sensitive compared with previously Au-S based nanoflares [57].

### 2.9. Gold Nanoparticles (AuNPs)

AuNPs have been widely used in biosensing due to their exceptional properties resulting in signal amplification and greater sensor performances, as previously discussed (*vide supra*). AuNPs have excellent electrical and catalytic properties, making them valuable components of electrochemical sensors, beyond their surface plasmon resonance properties; this makes them remarkable enhancers of the SPR electromagnetic field, resulting in signal amplification and higher sensitivity. Thus, AuNPs are widely applied in colorimetric sensors, and they are excellent fluorescence quenchers, enhancing the sensitivity and selectivity of fluorescence-based approaches. Their intrinsic biocompatibility, low cytotoxicity, and high stability in biological fluids make AuNPs ideal materials for biomedical applications, such as the detection of miRNAs in cancer cells, tissues, or biological fluids. For this reason, the use of AuNPs has significantly improved the performance of miRNA biosensors, highlighting their future use in clinical diagnosis [58,59,60,61,62].

An optical biosensing method for the detection and quantification of cancer-related miR-155, based on cross-linking AuNP aggregation, was designed by Esmaeili-bandboni et al. (2018). Citrate-capped AuNPs (18.7 ± 3.6 nm) were prepared and used to immobilise thiolated capture probes. Such a biosensing method was assessed in comparison to real-time PCR results, showing that the detection limit of 10 nM for this biosensor and PCR were approximately similar; however, the former method was simpler and faster than the previous ones [63].

For the determination of miR-141 in total RNAs extracted from human breast cancer cells (MDA-MB-231), Yu et al. (2018) designed a cascade-signal-amplification platform through the already-mentioned duplex-specific-nuclease (DSN)-assisted target-recycling, integrated with a catalytic hairpin assembly (CHA) reaction; this was further initiated by connector DNAs using hairpin-modified gold nanoparticles (HP-AuNPs) as the sensing unit. Using this platform, the formation of the AuNP network on the electrode for electrochemical and photoelectrochemical detection can occur. This method reached a detection limit as low as 25.1 aM (60 copies in a 4 μL sample) and excellent selectivity, to discriminate a single-base-mismatched sequence and other miRNAs [64].

Huang et al. (2019) developed a colorimetric and fluorescent dual-mode sensor for miRNA detection based on the optical properties of AuNPs and the duplex-specific-nuclease (DSN)-assisted signal amplification technique. AuNP surfaces were used to immobilize FAM-labelled hairpin probes (HPs) and to efficiently quench fluorescence due to the vicinity of the fluorophores. HPs could specifically hybridize with target miRNAs, and DSN could subsequently hydrolyse DNA strands in the DNA/RNA heteroduplexes, as already seen for other biosensors. The massive release of fluorophores into the solution results in fluorescence signal recovery. A large number of AuNPs were produced with short-chain DNA on their surfaces using the DSN-assisted signal amplification technique; these could aggregate in salt solution and participate in colorimetric detection. In this way a sensitive, accurate and selective detection method for miRNAs was developed and applied to the quantitative detection of miR-21, with a LOD as low as 50 pM and an optimal calibration range from 50 pM to 1 nM [65].

A different method was described by Hwu et al. (2019). They presented a strategy based on dark-field microwells, with a volume of 40 nL, for miRNA detection. This miniaturized device enables the readout of a gold nanoparticle assay without the need for a dark-field microscope. The method possesses great potential to widen the application of scattering-based nanoparticle assays for biological research and for point-of-care diagnostics. This procedure has been tested in both buffer solution and cell lysate. The authors demonstrated the feasibility of detecting miRNA in cell lysate with a DNA-AuNP assay. Additionally, this volume could be drastically decreased with further miniaturization, even to 1 nL, which means that, in combination with micro/nanopipetting technologies such as FluidFM, the dark-field microwells could be applied in single-cell sensing for which a small sample volume is required [66].

Wang et al. (2019) describe an improved lateral-flow assay, based on the use of catalytic hairpin assembly (CHA) and on signal amplification performed at the interface of AuNPs, for the detection of miRNA; this results in an assay that has a sensitivity improved by more than two orders of magnitude compared to its predecessors. In this study miRNA-21 was used as the target. The mechanism is similar to those we have already discussed: the presence of miRNA triggers the self-assembly of two hairpin DNAs into double-stranded DNA, and the display of biotin molecules on the surface of the AuNPs. The AuNPs carrying biotin are captured on the test line of the strip to show a red zone. This allows the visual recognition of miRNA without the need for any instrumentation. Fast quantification of miRNA via the red band’s intensity was carried out with the help of software, with a LOD of 0.89 pM. For the miRNA-21 assay, the enhanced lateral-flow assay was employed in cell extracts and spiked serum samples [67].

To simultaneously detect miRNA-21 and miRNA-200b, DNA-bridged assemblies of gold nanorods with upconverting nanoparticles (UPNPs), giving a generalized structure of AuNP@UCNP, together with a proper dye, were used by Qu et al. (2019) to exploit luminescence resonance energy transfer (LRET) and upconversion luminescence (UCL) intensity for effective miRNA quantification, reaching the outstanding LOD in the zeptomolar range [68].

Another methodological approach was developed by Canadya et al. (2019). They presented a highly specific strategy for a target miR sequence to provide the resolution of individual target molecules with high signal-to-noise ratio. Proper nucleic acid toehold probes were used to prepare AuNP tags that, when binding to a target miR sequence, displace a probe-protecting oligonucleotide and expose a capture sequence needed to selectively pull down the target-probe–nanoparticle complex to a photonic crystal (PC) biosensor surface. Photonic resonator absorption microscopy (PRAM) is a form of biosensor microscopy due to the reflected light intensity from the PC, which is dramatically and locally quenched by the presence of each individual nanoparticle when the surface plasmon-resonant wavelength of the nanoparticle tag matches the resonant wavelength of the PC nanostructure. Dynamic PRAM imaging of nanoparticle-tag capture leads to a LOD of 100 aM and sensitivity lower than 1 pM [69].

### 2.10. Miscellaneous Section

Several other nanosystems have been employed in the detection and quantification of miRNA, such as magneto-plasmonic nanoparticles (MPNPs) of the Fe_3_O_4_@Au MPNP kind, which exploited LSPR to detect a miR-375 cancer biomarker directly from unprocessed human serum with a 1 min response time, a LOD of 61.9 aM, a broad dynamic range (100 aM to 10 pM), and single-base-mismatch selectivity [70]. Polystyrene NPs were used to detect three colorectal-cancer-related miRNAs, miR-106a, miR-15a, and miR-21, with high sensitivity and low limits of detection [71,72].

Gold-loaded nanoporous superparamagnetic iron oxide nanocubes (Au-NPFe_2_O_3_NC) were applied in the electrocatalytic detection of miR-107 with a LOD of 100 aM and high reproducibility (% RSD = <5%, for *n* = 3) and specificity [73]. DNA mini-hexahedrons (DMHs) were suitable for intracellular miRNA detection, with fluorescence techniques that allowed the simultaneous detection of two cancer-related intracellular miRNAs; there were few false-positive signals, and it was able to discriminate healthy from cancerous cells [74]. Silver nanoparticles, in spite of their safety, relative biocompatibility, high versatility, and strong plasmonic properties, still have not found their niche in the field of miRNA biosensors. An example is given by Zhu et al. (2018) who used the surface-enhanced Raman spectroscopy (SERS) frequency-shift method for multiplex microRNA (miR-26a-5p, miR-223, miR-27a-3p) detection, in order to obtain an early serological diagnosis and discrimination between primary liver cancers in a patient cohort [75]. In Table 1, we resume the characteristics, described.

## 3. Cancer Treatment

As already described, miRNAs exert a critical role in cancer mechanisms. They are regulators in cancer onset and progression, and in the development of metastatic processes. For this reason, the inhibition of over-expressed miRNAs with an oncogenic role or the replacement of downregulated miRNAs that protect from carcinogenesis could be an interesting strategy to enhance anticancer therapies [76]. Nanomaterials could overcome the problems related to the delivery of miRNAs to the target cells. Traditionally, delivery systems for nucleic acids are of viral and non-viral origin. Nanocarriers are non-viral vectors that can improve drug stability, permitting and increasing their circulation time and their selective accumulation in cancer sites thanks to the enhanced permeability and retention effect and the presence of fenestrated blood vessels in tumours [77].

MiRNA delivery to cancer cells has been the topic of several reviews in the past few years; many authors have examined different nanotools that can be used for targeting and drug delivery, which can also be exploited to dispatch miRNA to neoplastic tissues. For instance, an article by Talluri et al. described the advantages and disadvantages of different lipid nanocarriers, such as liposomes, solid lipid nanoparticles, nanostructured lipid carriers, and lipid–polymer hybrid nanoparticles. The paper also examined the possibilities of using these technologies to treat breast cancer via the delivery of a chemotherapeutic agent. Opportunity is widely disclosed on targeted liposomal drug delivery [78]. Gallego et al. delved into treating the most common and aggressive CNS tumour, glioblastoma, and described ways to solve the main obstacle in the delivery of drugs to the brain area—overcoming the blood–brain barrier. They studied both invasive (intracerebral, intraventricular, and intracranial injections) and non-invasive (internal, parenteral) strategies, using previously illuminated forms of nanocarriers and the BBB transient opening method, which exploits focused ultrasound (FUS) at low acoustic pressures, in the presence of circulating microbubbles (MBs) [79]. Other authors turned their attention to the research on neuroblastoma, the most common extracranial solid cancer in children, which arises from nerve cells. The article focuses on the description of various miRNAs that could have a therapeutic effect for the treatment of neuroblastoma, with a description of individual miRNAs, their targets, and models on which they were tested. Additionally, a separate chapter is devoted to the definition of approaches and nanotechnology-based formulations, which include complexation, encapsulation, and conjugation-based strategies, either by passive, active, or stimuli-response-based models. The study also describes circulating microRNAs used to diagnose neuroblastoma [80].

Given the large number of articles that have appeared in the literature during the past few years concerning nanosystems used in miRNA delivery and therapeutic strategies against cancer, part of this review will be focused on the studies carried out from 2016 to 2022, and the latest research advances in this field.

Nanocarriers can be made of different materials, both organic and inorganic, and polymers. A vast assortment of nanomaterials are used for the delivery of miRNAs, e.g., Tiram et al. (2016) synthesized an aminated polyglycerol dendritic nanocarrier (dPG-NH2) and designed dPG–NH2–microRNA polyplexes to target osteosarcoma. The nanocarrier vehiculated miR-34a, miR-93, and miR-200c, which diminished in the fast-growing angiogenic phenotype. The treatment with microRNAs using dPG-NH2 significantly prolonged the inactivity period of osteosarcomas in vivo [81]. Sun et al. (2017), in turn, created a nanovector composed of mesoporous magnetic clusters conjugated with ternary polymers, aimed at efficient in vivo miRNA delivery. The dispatch of miR-100 to cancer cells permitted cell-proliferation inhibition, inducing apoptosis in vitro. Moreover, the combination of an miR-100 nanovector with conventional chemotherapy showed an ability to specifically suppress tumour growth [82]. Ma et al. (2020) reported pH/ATP-activated nanocomplexes for increasing the cytosolic delivery of miR146a, which can effectively inhibit the expression of epidermal growth-factor receptor (EGFR) in androgen-independent prostate cancer (AIPC). The nanocomplexes described by these authors show their capability to suppress the growth of the tumour after five weeks of treatment in in vivo experiments [83]. Javanmardi et al. (2018) used a series of branched polyethylenimine (PEI) modifications including PEGylation (PEG2k-PEI) for steric shielding, and redox-sensitive crosslinking for synthesizing PEG2k-PEI-ss nanogels to deliver anti-miR-21. The authors studied the effect of this nanomaterial against the A2780 ovarian cancer cell line in terms of the loading ability of anti-miR-21, the formation of stable nanocomplexes, cellular delivery, and transfection efficiency, discovering the ability of these nanogels to load and preserve anti-miR-21 in vitro [84]. Nagachinta et al. (2020) developed biocompatible nanosystems based on sphingomyelin (SM), with electrostatic interactions with stearylamine, to specifically deliver miRNA145 to combat colorectal cancer. The system showed strong anticancer activity with suppression of tumour proliferation, colony forming, and migration capability [85]. Elfiky (2021) recently described the use of hyperbranched polyamidoamine synthesized via a one-pot reaction, followed by decoration with lactobionic acid (LA-PAMAM), as a carrier to vehiculate miRNAs against hepatocellular carcinoma (HCC). In fact, MiR-218 is a tumour suppressor down-regulated in HCC that the authors successfully delivered to selectively suppress HCC, in vitro, against HepG2 cells; moreover, it reduced tumour development in vivo in mice [86].

The previous examples represent just a narrow gamut of the nanosystems used to vehiculate miRNAs to tumour sites, since the nanomaterials described above were not the most commonly tested in the last few years. Thus, in the following sections, we report the most commonly used nanomaterials, the miRNAs delivered, and their specific targets.

### 3.1. Extracellular Vesicles

Extracellular vesicles (EVs) are composed of a lipidic bilayer membrane secreted from cells. EVs, which include exosomes and micelles, are present in body fluids and permit communication and the transport of materials [87]. EVs are natural nanocarriers, varying in size from 100 to 1000 nm. They form via the outward budding of the plasma membrane domains, and are secreted by cells into the extracellular environment in the form of exosomes, micro-vesicles, or apoptotic bodies. Bose et al. (2018) described the use of tumour-cell-derived extracellular vesicles (TEVs) for cancer-targeted delivery of therapeutics and theranostics, in order to create a nanoplatform for tumour detection and treatment. Thus, they developed a TEV-based nanoplatform for multimodal miRNA delivery and phototherapy treatments by dispatching anti-miR-21 against HepG2 and SKBR3 cells in vitro, and causing a TEV-mediated accumulation of anti-miRNA-21 in tumours in vivo [76]. Many other authors in the last few years used EV to deliver miRNAs for targeted therapy against cancer, as described below.

### 3.2. Exosomes

Exosomes, nano-sized cell-derived vesicles of 40–100 nm in size, have been employed as non-synthetic carriers of various pharmaceutics in numerous studies, also regarding cancer. They are generated through endocytosis and are carriers of information among different cell types. Interestingly, exosomes have a specific protein on their surface that permits the identification of the targeted cell [88].

Naseri et al. (2018) demonstrated that exosomes isolated from bone-marrow-derived mesenchymal stem cells (MSCs-Exo) can efficiently deliver anti-miR-142-3p to reduce the miR-142-3p and miR-150 levels in breast cancer cell lines. The targets of miR-142-3p and miR-150 are associated with the clonogenic and tumourigenic features of breast cancer stem cells (BCSCs) and are implicated in hyperproliferation of cancer cells in vitro, and mammary glands in vivo. Authors also evaluated the in vivo distribution of the MSCs-Exo in tumour-bearing mice, showing that MSCs-Exo are able to penetrate the tumour site and are suitable nanovehicles for these purposes [89]. Additionally, Vakhiteh et al. (2020) vehiculated miRNAs against breast cancer cells. They modified exosomes derived from genetically modified dental-pulp MSCs (DPSCs), prepared using XMIRXpress-34a lentivectors, as a carrier to deliver tumour suppressor miR-34a against breast carcinoma cells [90]. More recently, Moradi-Chaleshtori (2021) delivered miR-130 to M2 macrophages. Macrophages are already known to assist the malignant phenotype of tumour influencing growth, angiogenesis, metastasis, and drug resistance. The authors treated these cells using tumour-derived exosomes, triggering upregulation of specific markers and cytokines, and downregulation of specific M2 markers enhancing phagocytosis. This study demonstrated that such macrophages reprogrammed with the nanodelivery system regulate tumour invasion and metastasis in co-cultured breast cancer cells [91]. Moreover, Yang et al. (2021) described a novel method to produce a high number of exosomes with cellular nanoporation. These exosomes, containing therapeutic mRNAs and targeting peptides, were prepared by transfecting various source cells with plasmid DNAs and stimulated with a focal and transient electrical stimulus, promoting the release of exosomes carrying transcribed mRNAs. This innovative method permitted an up to 50-fold increase in the production of exosomes and more than a 103-fold increase in exosomal mRNA transcripts when compared to other exosome-production strategies. They successfully tested the efficiency of the liposomes created in a glioma mouse model, enhancing tumour growth inhibition [92].

### 3.3. Micelles

Micelles have shown great potential in cancer therapy. The size of micelles favours the enhanced permeability and retention effect, while avoiding macrophage response. Yin et al. (2019) reported the targeted delivery of anti-miRNA to cancers using RNA micelles created on a scaffold of phi29-packaging RNA and carrying oligonucleotides composed of 8nt locked nucleic acid complementary to microRNA21 (miR-21). The resulting micelles carrying anti-miR-21, specifically directed to cancer cells, showed strong binding and internalization ability, and induced cell apoptosis in vitro. Moreover, animal trials described in the same paper revealed tumour inhibition in xenograft models [93]. Li et al. (2017) used a three-layered polyplex with folic acid to deliver miR-210 into breast cancer cells, causing growth inhibition [94,95].

### 3.4. Niosomes

Niosomes are a class of biodegradable cationic lipid-based vesicle made of synthetic nonionic surfactants, usable to deliver high concentrations of drugs for target therapy in tumour sites (Hemati et al., 2019). A recent study by Ghaffari et al. (2021) disclosed the potential of specific cationic niosomes containing the tween60-cholesterol-DOTAP-DSPE-PEG2000 composite to vehiculate miR-15a and miR-16–1 and regulate the expression of the Bcl-2 gene in the modulation of apoptosis rate in prostate cancer cells. Experiments in vitro have been carried out by transfecting PC3 cells with nanocarriers, leading to a significant decrease in the expression of the Bcl-2 gene and an increase in the degree of cell death in PC3 cells compared with other treatment groups [96].

### 3.5. Nanoparticles

Nanoparticles are employed in target therapy for their potential in the treatment of several target cancer cells, and to enhance the accumulation of the delivered molecules at the tumour site. Moreover, the hyperpermeability of blood vessels in cancer masses allows nanoparticles to enter into intercellular spaces and flow into the tumour [88]. Some recent studies have successfully used nanoparticles to vehiculate miRNAs in cancer tissues. Nanoparticles were employed against prostate cancer in a mouse model. Binzel et al. (2016) reported the application of a thermodynamically ultra-stable three-way junction of the pRNA of a phi29 DNA-packaging motor for specific targeting and treatment of prostate cancer cells. RNA nanoparticles vehiculated anti-miR17 or anti-miR21 as therapeutics. These systems permitted maintenance of the stability of RNAs after systemic injection in mice, a strong and specific bond to the tumour, and reduced accumulation in other organs. Moreover, this system repressed tumour growth at low doses with high efficiency in vivo [97].

Li et al. (2017) developed a combined therapy of miRNA-21 antisense oligonucleotides (ASO-miR-21) and gemcitabine using targeted co-delivery polyethylene glycol–polyethylenimine–magnetic iron oxide nanoparticles; they studied its synergistic inhibitory effects on pancreatic cancer cells, resulting in the suppression of a epithelial–mesenchymal transition, clone formation, migration, and the invasion of pancreatic cancer cells in vitro. Moreover, animal tests using these nanoparticles induced potent inhibition of tumour proliferation and metastasis [98]. Yoo et al. (2017) investigated the use of nanoparticles to treat metastatic breast cancer at stage IV. Authors reported the inhibition of miRNA-10b-a, which regulated the metastatic process, using magnetic nanoparticles vehiculating anti-miRNA-10b (MN-antiR10b). These nanoparticles were injected intravenously in mice implanted with murine 4T1 cells to create metastases. The nanoparticles accumulated selectively in tumour cells, causing the regression of metastasis after one week of treatment in combination with doxorubicin, and also reducing mortality [99]. Another recent investigation explored the use of glutathione-responsive chitosan-thiolated-dextran (TD-miR)-conjugated miR-145 nanoparticles, targeted with anti-nucleolin aptamer AS1411 for cancer treatment. In vitro studies indicated the capability of the device as a delivery system to preserve and transfect the cargo and release the delivered miRNA into the cytoplasm, resulting in gene silencing and cancer cell apoptosis [100]. Vandghanooni et al. (2018) described the fabrication and use of anti-nucleolin aptamer-decorated PEGylated poly (lactic-co-glycolic acid)-nanoparticles containing cisplatin (Ap-CIS-NPs) and anti-miR-21 (Ap-anti-miR-21-NPs) against ovarian cancer cells. In fact, the overexpression of miRNA-21 is associated with cisplatin resistance, and these experiments permitted the sensitization of the cells to this drug, enhancing their mortality [101]. Other authors developed bioreducible poly (beta-amino ester)-nanoparticles with high intracellular delivery efficacy and low cytotoxicity, also combining cancer-stem-cell-inhibiting miRNAs to create polymeric nanoparticles containing miRNA (nano-miRs) to treat glioblastoma. Lopez-Bertoni et al. (2018) showed that these nano-miRs are able to deliver miRNA and inhibit the growth of human glioblastoma cells, enhancing, at the same time, the response to γ–radiation. Moreover, the co-delivery of miR-148a and miR-296-5p within the nano-miR particles permitted long-term survival from glioblastoma in a mouse model [102]. Panebianco et al. (2019) used silica dioxide nanoparticles (SiO2NPs) as the carrier in the delivery of miR-34a toward breast cell lines and primary culture cells in a 3D model, leading to reduced mammary tumour growth and suggesting the capability of silica nanoparticles to deliver miR-34a in mammary tumour sites [103]. Sukumar et al. (2019) explored the possibility of using polyfunctional gold–iron oxide nanoparticles (polyGIONs) to overtake the brain barriers and target glioblastoma (GBM). To prepare the nanosystem, the polyGION’s surface was loaded with healing miRNAs (miR-100 and antimiR-21) and then administered to GBMs in mice. These nanoformulations allowed the sensitization of GBM cells to the classical chemotherapy drug temozolomide [104]. Chiang et al. (2019) described the delivery of miR-29b to chronic lymphocytic leukemia cells using lipid-complex nanoparticles, leading to the downregulation of DNMT1 and DNMT3A, the modulation of DNA methylation, and increasing p21 expression in vitro and in vivo in mice. The results of the treatment showed that it permitted the reprogramming of cell-cycle regulators with decreased SP1 and increased p21 expression, leading to cell-cycle arrest and also to survival in vivo [105]. Perepelyuk et al. (2018) evaluated the therapeutic efficacy and pharmacokinetics of mucin1-aptamer-functionalized miRNA-29b-loaded hybrid nanoparticles (MAFMILHNs) to counteract lung cancer in mice. MAFMILHNs, fabricated with isoelectric point-based nanotechnology, were able to downregulate oncoprotein DNMT3B both in vitro and in vivo, selectively, in tumour cells and tissues; this resulted in the inhibition of tumour growth in mouse models [106]. Furthermore, Qian et al. (2018) reported the capability of plasmonic gold nanoparticles (GNPs) combined with miR-21 to control the exocytosis process in miRNA-21-positive MCF-7 breast cancer cells; this highlighted the rapid accumulation of GNPs in the presence of intracellular miR-21 and the inhibition of exocytosis of the complex. Interestingly, the authors found that the system was intracellularly maintained for 24h. Moreover, the use of a 680 nm (near infrared region) laser in the presence of these nanogold–miRNA complexes permitted killing of the MCF-7 cells, indicating good potential of such nanocarrier systems in cancer treatment [107].

Chen et al. (2019) developed UiO-68 metal–organic framework nanoparticles (NMOFs) loaded with doxorubicin and locked via the method of a structurally engineered duplex nucleic acid framework; these included the complementary sequence to the microRNAs miR-21 or miR-221, specific miRNA biomarkers for MCF-7 breast cancer cells and OVCAR-3 ovarian cancer cells, respectively. In vitro experiments revealed that the respective NMOFs are unlocked by miR-21 or miR-221, resulting in selective cytotoxicity toward the specific targets—MCF-7 breast cancer cells or OVCAR-3 ovarian cancer cells—thus supporting the hypothesis that two different miRNAs can sustain the release of two different drugs targeting two different types of cancer cells [108]. A more complex system of delivery is the stimuli-responsive hybrid structures composed of drug-loaded UiO-66 metal–organic framework nanoparticles, NMOFs, locked by DNA tetrahedra gates presented by Zhang et al. (2021). The hybrid systems described merge the loading capability of drugs in the cell permeation properties of the DNA tetrahedra. The nucleic acid-functionalized UiO-66 NMOFs loaded with miR-21 or miRNA-155 were used to release the drugs in the target cancerous area [109].

Another target for nanomaterials with miRNAs is triple-negative breast cancer (TNBC), characterized by CD24, CD44, CD133, ALDH1, and ABCG2 markers. Yin et al. (2019) applied nanotechnologies to deliver anti-microRNA (miRNA) for TNBC therapy using the three-way junction (3WJ) as the scaffold, to carry an RNA aptamer binding to a CD133 receptor, to inhibit miRNA21. This study revealed the specific uptake of the nanoparticles to breast cancer stem cells (BCSCs) and TNBC cells also reduced cell migration both in vitro and in vivo without inducing an immune system response [110]. Other authors developed a successful application of HA-Dual miRNA NPs against TNBC. It is already known that miR-34a is downregulated in TNBC and miR-10b is upregulated, promoting tumourigenesis and metastasis. In this regard, Ahir et al. (2020) delivered anti-miR-10b and tumour-suppressive miRNA-34a into TNBC cells for an improved efficacy of chemotherapy using tailored mesoporous silica nanoparticles (MSNs), for the co-delivery of miR-34a-mimic and antisense-miR-10b. Moreover, they were coated with a hyaluronic acid-appended PEG-PLGA polymer for specific targeting of tumour cells in the animal trial model [111]. Additionally, Unal et al. (2020) developed AGO2-conjugated nanoparticles to target miRNAs against breast cancer. The authors used superparamagnetic iron oxide (Fe_2_O_3_) nanoparticles (SPIONs, SP) to vehiculate microRNA (MIR376B) into HER2-positive breast cancer cell lines in vitro and in vivo, combining the nanocarrier with cisplatin. The resulting combination of cisplatin and MIR376B-loaded nanoparticles increased the treatment efficacy both in vivo and in vitro, acting selectively on HER2-positive breast cancer cells and in the tumour site [112].

Upadhyay et al. (2019) formulated PEGylated PLGA thymoquinone nanoparticles (TQ-Np) for improved TQ delivery to non-small-cell lung carcinoma cells. Non-small-cell lung carcinoma A549 cells over-express a transferrin receptor that specifically bind transferrin (TF). For this reason, the authors developed a decoration of the PEGylated PLGA thymoquinone nanoparticles using transferrin (TF-TQ-Np) to target the nanoparticles specifically on tumourigenic cells, enhancing their internalization to control the p53/miR-34a/miR-16 axis [113].

In a recent study, Shi et al. (2019) significantly induced cell-cycle arrest in G1 by using functionalized upconversion SiO_2_-coated nanoparticles (UCNP) as the vector with polyetherimide (PEI) and miR-145, functionalized via hyaluronic acid. These nanoparticles induced CCND1, CDK6 and CCNE2 protein downregulation in vivo, inhibiting tumour growth in a mouse colon cancer model [114]. Shao et al. (2020) synthesized flower-shaped SiO₂-PEI nanoparticles with a miR-let-7c-5p-expressed plasmid, which was able to transfer miR-let-7c-5p to human epithelial carcinoma (HeLa) cells. Flower-shaped SiO₂-PEI nanoparticles with miR-let-7c-5p are able to suppress cervical cancer [115]. Moreover, Liu et al. (2021) created stabilized polymeric nanoparticles for the co-delivery and release of miR-124 and anti-miR-21, regulating the tumourigenesis- and angiogenesis-signalling pathways of treated glioblastoma cells, and consequently, resulting in the inhibition of tumour progression [116].

Maghsoudnia et al. (2020) designed and fabricated the nanocarriers let-7b-PAMAM (G5)-TPP and let-7b-PAMAM (G5)-TPP-HA, which are able to deliver let-7b miRNA to lung cancer cells to target their mitochondria. Let-7b-loaded nanoparticles showed the ability to significantly reduce cell viability by inducing apoptosis [117]. In another recent study, Wang et al. (2021) developed novel RNA nanoparticles based on a 6-way junction (6WJ), allowing the conjugation of three liver-targeting ligands, one copy of miR122, and 24-copies of the paclitaxel drug. The authors chose miR122 because it is downregulated in tumours, and is able to inhibit the expulsion of the delivered drugs. The in vivo studies on xenografted mice showed that the RNA nanoparticles predominantly accumulated in the tumour and efficiently inhibited tumour growth after 22 days of administration [118]. Finally, a particularly interesting kind of nanoparticle was created by Zhou et al. (2021). In fact, they prepared nanozymes, which are particular nanomaterials with enzyme-like properties, such as cerium oxide (CeO_2_) nanoparticles, that perform excellent activities. The authors integrated these CeO_2_ nanozymes with miR-181a to increase the effect of radiotherapy against oesophageal cancer, alleviating hypoxia [119].

### 3.6. Nanoplatforms

Nanoplatforms are platforms created using nanoparticles, with the purpose of enhancing the efficacy of the carrier in therapeutic applications by increasing their stability. Kim, H.Y. et al. (2017) developed a fluorescence-switchable theragnostic nanoplatform using hyaluronic acid (HA)-conjugated graphene oxide (GO), to exploit two different functions: sensing oncogenic miR-21 and inhibiting its tumourigenicity. The experiments, performed in breast cancer model mice demonstrated the capability of the nanoplatform to target breast cancer cells, visualize the endogenous miR-21, and reduce its tumourigenicity [120]. Assali et al. (2018) vehiculated miR-101, an intense cancer suppressor targeting and suppressing the Stathmin1 protein; it induced apoptosis and cellular stress. The authors used graphene oxide (GO) nanoplatform sheets bound with polyethylene glycol and poly-l-arginine to enhance miRNA target delivery. The experiments revealed that the nanoplatforms were able to increase internalization by enhancing trafficking in the cell membrane, and facilitated endosomal scape, improving selective transfection in cancer cell lines (MCF7, MDA-MB-231) in comparison with non-cancerous cells [121]. In another study, the authors used multifunctional nanoplatforms to deliver miR-101 and NIR thermal therapy, in order to induce apoptosis in breast cancer cells. Specifically, the authors used Au nanorods or nanospheres covered with graphene oxide. Nanoplatforms indicated better performance in cellular uptake and gene transfection than spherical ones, and NIR thermal therapy, synergically with miR-101, activated the apoptotic pathway, significantly decreasing breast cancer cell viability [122,123]. In Table 2, we evaluate miRNAs for therapy.

## 4. Rationale behind Article Selection

**Figure 3:**
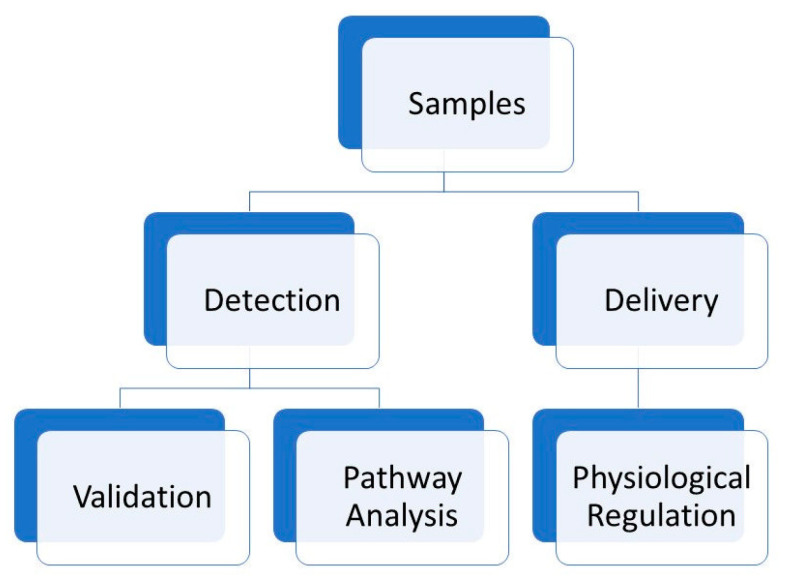
Summary of methodological workflow for studying miRNA function. The workflow summarizes the emerging high-throughput experimental approaches for the study of miRNA gene regulatory networks.

An electronic search was performed using Medline databases (PubMed interface) for articles published from 2011 to 14 February 2022 (10-year interval). The terms used for the search were “miRNA AND nanotechnologies”, or “Nano Mirna in detection” or “Nano Mirna in treatment”, or “Diagnostic and therapeutic role of microRNAs in nanotechnology”. The inclusion criteria were (a) full-text articles reporting studies on a miRNA of interest in vitro or in vivo; (b) articles written in English; (c) meta-analyses with data on that topic; and (d) studies approved by an ethics committee and performed in accordance with the principles of the Declaration of Helsinki. The electronic search yielded 272 results. After the exclusion of duplicates, irrelevant articles, reviews, papers not written in English, or papers with missing data, 116 articles were included for review. Abstracts were selected, and the chosen full-text articles were reviewed. This review is based on previously performed studies, and contains no new studies with human or animal participants performed by any of the authors (Figure 3).

## 5. Conclusions

MiRNAs represent a novel tool for disease detection and treatment. The regulation of specific gene expression and the modification of cellular function can be accomplished by targeting specific miRNA expression. MiRNAs can be chemically modified and can be efficiently employed in medical fields, together with nanotechnologies; this represents a novel developing area of research, which should still be completely disclosed. In this review we reported on several specific methodologies for the delivery of miRNA and different nanoparticle-based detection procedures. In fact, from the analysis of the literature from the past few years, it emerges that miRNAs can play an important role as valuable biomarkers for cancer detection; moreover, their levels can be revealed—with LODs in the range of nano-, pico- or femto-molar concentrations and often with high specificity—via the application of proper metal-based nanodevices and exploitation of their SPR to give colorimetric responses. Trends in this field of research also show that graphene and its derivatives, quantum dots, and other nanomaterials can be used to assemble sensitive biosensors in the detection of miRNAs, and that electrochemical biosensors are able to reach very low detection limits, down to the atto range. Moreover, miRNAs in association with different nanocarriers can be used in target cancer therapy, as demonstrated by the large number of articles that appeared in the literature on this topic. The nanovectors involved can be very different in nature, since they can be made of either organic or inorganic nanomaterials, with very specific properties and characteristics. Extracellular vesicles (e.g., exosomes, micelles, and niosomes), nanoparticles, nanoplatforms, etc.—often associated with other chemotherapeutic drugs such as cisplatin—have been employed to transport miRNAs to increase their efficacy. The results seem to be very encouraging and demonstrate the interest and great activity of researchers in this field.

We are, thus, able to state that, through the interchange of several branches of science such as medicine, biotechnology, synthetic biology and nanotechnology, the miRNA–nanotechnology combination could allow the detection of different novel biomarkers while offering contemporary novel treatments to cure diseases. However, nanomedicine is still an emerging area of research, and its applications in disease detection and protection still represent a challenge. Nanotechnology-assisted strategies, together with miRNAs, could pave the way for future integrated clinical management.

## Figures and Tables

**Table 1:** miRNAs for diagnosing.

Type of Biosensor/Sensing System	Detected miRNAs/miRNA Precursors	Detection Limit	Types of Samples	References
Colorimetric Biosensor	let-7d	ca. 1.06 nM	--	[16]—Park Y et al. (2018)
	miR-148a	~1.9 nM	Cancer cells	[18]—Ye C et al. (2017)
		~1.9 nM	Cancer cells	[19]—Cai J et al. (2019)
	miR-21, miR-155	<1 ng/µL	Cancer cell lines	[12]—Mollasalehi H et al. (2021)
Surface plasmon resonance (SPR)	miR-141	1 fM	Cell extractions	[24]—Wang et al. (2016)
		0.5 fM	--	[28]—Nie et al. (2017)
		0.6 fM	--	[27]—Liu et al. (2017)
	miR-21	1 pM	Human total RNA	[26]—Li et al. (2016)
	let-7a	0.8 fM	Human serum and cell lysate	[29]—Wenyan Nie et al. (2018)
	miR-148a	~1.9 nM	--	[19]—Jun Cai et al. (2020)
	miR-210	0.78 nM	--	[30]—Portela et al. (2020)
Graphene	miR-29a, miR-144	0.05 pmol	Human serum and HeLa cells	[34]—Treerattrakoon et al. (2019)
	miR-21, miR-1246, miR-Let7b	10 fM	Urine	[34]—Kim et al. (2020)
Carbon dots	miR-155	0.1 aM	MCF-7 and human serum	[37]—Mohammadi et al. (2018)
	miR-21	0.3 nM	--	[38]—Mahani M et al. (2019)
		0.03 fM	MCF-7	[39]—Mohammadi S et al. (2021)
Molecular beacon	miR-21, miR-122, miR-155	--	Cell lines	[41]—Wang et al. (2021)
	miR-155-5p	--	--	[42]—Kim et al. (2021)
Electrochemical biosensor	miR-155	0.6 fM	--	[43]—Azimzadeh et al. (2016)
	miR199a-5p	4.5 fM	--	[44]—Ebrahimi et al. (2018)
	microRNA	1.2 aM	--	[67]—Cheng et al. (2019)
	miR-141	7.78 fM	--	[46]—Zhou et al. (2020)
	miR-155	20 zmol	--	[47]—Hakimian et al. (2020)
		0.15 fM	Human serum	[48]—Yazdanparast et al. (2020)
	miR-21	0.020 fM	--	[49]—Pothipor et al. (2021)
	miR-21, miR-155, and miR-210	--	--	[50]—Pothipor C et al. (2021)
	miR-21	0.14 U/mL	--	[51]—Pothipor C et.al (2022)
Förster resonance energy transfer (FRET)	miR-21	--	Cancer cells	[53]—Li et al. (2021)
	miR-148	42 fM	Human cancer- and normal-cell lines	[54]—Wang et al. (2018)
Magneto-plasmonic Nanoparticle	miR-375	61.9 aM	Human serum	[70]—Che C et al. (2022)
Nanoflares	miR-21, miR-141	--	Cancer cells	[55]—Li J et al. (2018)
	miR-375	0.36 fM	Human serum	[56]—Zhao et al. (2020)
	miR-21	--	Living cells	[57]—Qing Z et al. (2020)
Polystyrene nanoparticles	miRNAs, miR-106a, miR-15a, and miR-21	--	--	[71]—Wang et al. (2020)
Iron oxide nanocubes	miR-107	100 aM	--	[73]—Nazmul et al. (2018)
DNA mini hexahedron (DMH) and DNA-based probe	miR-21, miR-1246	--	Healthy and cancerous cells	[74]—Dong et al. (2019)
Gold nanoparticles (AuNPs)	miR-155	10 nM	--	[63]—Esmaeili-bandboni (2018)
	miR-141	25.1 aM	--	[64]—Yu et al. (2018)
	miR-21	50 pM	--	[65]—Huang et al. (2019)
	microRNA	--	Cell lysate	[66]—Hwu et al. (2019)
	miR-21	0.89 pM	Cell extracts and serum samples	[67]—Wang et al. (2019)
	miR-21, miR-200b	Zeptomolar range	--	[68]—Qu et al. (2019)
Silver nanoparticle (AgNFs)	miR-26a-5p, miR-223 and miR-27a-3p	--	--	[75]—Zhu et al. (2018)

**Table 2:** miRNAs for Therapy.

Type of Nanocarrier	Target miRNAs	Target	References
Nanoparticles	MiR-17, miR-21	Prostate cancer cell	[97]—Binzel D et al. (2016)
	miR-21	Pancreatic cancer cell	[98]—Li Y et al. (2017)
	MiR-10b-a	Tumour	[99]—Yoo B et al. (2017)
	miR-145	Cancer	[100]—Tekie F et al. (2018)
	miR-21	Ovarian cancer cells	[101]—Vandghanooni S et al. (2018)
	miR-148a, miR-296-5p	Glioblastoma cells	[102]—Lopez-Bertoni H et al. (2018)
	miR-34a	Breast cancer cells	[103]—Panebianco F et al. (2019)
	miR-100, miR-21	Glioblastoma	[104]—Sukumar U et al. (2019)
	miR-29b	Leukaemia	[105]—Chiang C et al. (2019)
	miR-29b	Lung cancer	[106]—Perepelyuk M et al. (2018)
	miR-21	Breast cancer	[107]—Qian R et al. (2018)
	miR-21, miR-221	Breast cancer	[108]—Chen W et al. (2019)
	miR-21, miR-155	Cancer	[109]—Zhang P et al. (2021)
	MiR-21	Triple-negative breast cancer	[110]—Yin H et al. (2019)
	miR-34a, miR-10b	Triple-negative breast cancer	[111]—Ahir M et al. (2020)
	miR-376b	HER2-positive breast cancer	[112]—Unal O et al. (2020)
	miR-34a, miR-16	Non-small-cell lung cells	[113]—Upadhyay P et al. (2019)
	miRNA-145	Colon cancer cells	[114]—Shi H et al. (2019)
	miR-let-7c-5p	HeLa cells	[115]—Shao L et al. (2020)
	miRNA-124, miRNA-21	Glioblastoma cells	[116]—Liu Y et al. (2021)
	miR-let-7b	Lung cancer cells	[117]—Maghsoudnia N et al. (2020)
	miR-122	Tumour cells	[118]—Wang H et al. (2021)
	miR-181	Oesophageal cancer	[119]—Zhou X et al. (2021)
Nanoplatforms/nanosystems	miR-21	Breast cancer cells	[120]—Kim H et al. (2017)
	miR-101	MCF7, MDA- MB-231	[121]—Assali A et al. (2018)
	MiR-101	Breast cancer cells	[122]—Assali A et al. (2018)
		Breast cancer cells	[123]—Luo Y et al. (2021)
Extracellular vesicles	miR-21	SKBR3 cells	[76]—JC Bose R et al. (2018)
Exosomes	miR-142-3p, miR-150	Breast cancer cells	[89]—Nseri Z et al. (2018)
	miR-34a	Breast cancer cells	[90]—Vakhshiteh F et al. (2021)
	miR-130	Breast cancer cells	[91]—Moradi-Chaleshtori M et al. (2021)
Micelles	miR-21	Cancer cells	[93]—Yin H et al. (2018)
	miR-210	Breast cancer cells	[95]—Li Y et al. (2017)
Niosomes	miR-15a, miR-16-1	Prostate cancer cells	[96]—Ghaffari M et al. (2021)

## Abbreviations

3-QD@DNA NC	Triple-CdTe quantum-dot-labelled DNA nanocomposites
3SPCE	Three-screen-printed carbon electrode
AgNCs	Silver nanoclusters
AIPC	Androgen-independent prostate cancer
Au-NPFe2O3NC	Gold-loaded nanoporous superparamagnetic iron oxide nano-cubes
AuNPs	Gold NPs
AuNPs/GQDs/GO	Gold nanoparticles/graphene quantum dots/graphene oxide
AuNPs-MoS2	Gold-nanoparticle-decorated molybdenum sulfide
BCSCs	Breast cancer stem cells
BHQ1	Black Hole Quencher 1
BRCA1	Breast cancer 1 gene mutation
CA 15-3	Cancer antigen 15-3
C-dots	Carbon dots
CESA	Cyclic enzymatic signal boost
CHA	Catalytic hairpin assembly
CQDs	Carbon quantum dots
CS	Nanoparticles of chitosan
CS-MB	Chitosan-molecular beacon
d PG-NH2	Polyglycerol dendritic nanocarrier
DMHs	DNA mini hexahedrons
DPSCs	Dental pulp MSCs
DPV	Differential pulse voltammetry
DSA	Double signal amplification
DSN	Double-strand specific nuclease
DTPs	DNA tetrahedron probes
DTPs-Au	DNA tetrahedron probes onto gold films
EATR	Enzyme-assisted target recycling
EGFR	Epidermal growth-factor receptor
EVs	Extracellular vesicles
FC60	Fullerene nanoparticles
FRET	Förster or fluorescent resonance energy transfer
FUS	Focused ultrasound
GBM	Glioblastoma
GCE	Glassy carbon electrode
GNPs	Plasmonic gold nanoparticles
GNRs	Gold nanorods
GO	Graphene oxide
GO–AuNPs	Graphene oxide–gold nanoparticles
GP	Nanocomposite of graphene
GQDs	Graphene quantum dots
HA	Hyaluronic acid
HCC	Hepatocellular carcinoma
HP-AuNPs	Hairpin-modified gold nanoparticles
HPs	Hairpin probes
LOD	Limit of detection
LRET	Luminescence resonance energy transfer
LSPR	Surface plasmon resonance
MB	Molecular beacon
MBs	Microbubbles
MCF-7	Breast cancer cells
miRNA	microRNA
MPNPs	Magneto-plasmonic nanoparticles
MSCs-Exo	Exosomes from bone-marrow-derived mesenchymal stem cells
MSNs	Mesoporous silica nanoparticles
nano-miRs	Nanoparticles containing miRNA
NGS	Next-generation sequencing
NMOFs	Metal–organic framework nanoparticles
NPs	Nanoparticles
PC	Photonic crystal
PEG2k-PEI	Polyethylenimine (PEI) modifications including PEGylation
PEI	Polyethylenimine
PEI	Polyetherimide
PolyGIONs	Polyfunctional gold–iron oxide nanoparticles
PPY	Polypyrrole
PRAM	Photonic resonator absorption microscopy
QDs	Quantum dots
qRT-PCR	Real-time polymerase chain reaction
RCA	Rolling-circle amplification
RNAP	RNA probes
RSV	Resveratrol
SA-aptamer	Streptavidin aptamer
SERS	Surface-enhanced Raman scattering
SiO2NPs	Silica dioxide nanoparticles
SM	Sphingomyelin
SPCE	Screen-printed carbon electrode
SPIONs, SP	Superparamagnetic iron oxide nanoparticles
SPR	Surface plasmon resonance
ssDNA	Single-stranded DNA
SWV	Square-wave voltammetry
TEVs	Tumour-cell-derived extracellular vesicles
TF	Transferrin
TF-TQ-Np	Thymoquinone nanoparticles using transferrin
TMSD	Toehold-mediated strand displacement
TNBC	Triple-negative breast cancer
TQ-Np	Thymoquinone nanoparticles
UCL	Upconversion luminescence
UPNPs	Gold nanorods with upconverting nanoparticles
